# Distinguishing patterns in the dynamics of long-term medication use by Markov analysis: beyond persistence

**DOI:** 10.1186/1472-6963-7-106

**Published:** 2007-07-10

**Authors:** Tanja T Menckeberg, Svetlana V Belitser, Marcel L Bouvy, Madelon Bracke, Jan-Willem J Lammers, Jan AM Raaijmakers, Hubert GM Leufkens

**Affiliations:** 1 Division of Pharmacoepidemiology & Pharmacotherapy, Utrecht Institute for Pharmaceutical Sciences (UIPS), Utrecht, The Netherlands; 2 SIR Institute for Pharmacy Practice and Policy, Leiden, The Netherlands; 3 Department of Pulmonary Diseases, Heart Lung Centre Utrecht, University Medical Centre, Utrecht, The Netherlands

## Abstract

**Background:**

In order to accurately distinguish gaps of varying length in drug treatment for chronic conditions from discontinuation without resuming therapy, short-term observation does not suffice. Thus, the use of inhalation corticosteroids (ICS) in the long-term, during a ten-year period is investigated. To describe medication use as a continuum, taking into account the timeliness and consistency of refilling, a Markov model is proposed.

**Methods:**

Patients, that filled at least one prescription in 1993, were selected from the PHARMO medical record linkage system (RLS) containing >95% prescription dispensings per patient originating from community pharmacy records of 6 medium-sized cities in the Netherlands.

The probabilities of continuous use, the refilling of at least one ICS prescription in each year of follow-up, and medication free periods were assessed by Markov analysis. Stratified analysis according to new use was performed.

**Results:**

The transition probabilities of the refilling of at least one ICS prescription in the subsequent year of follow-up, were assessed for each year of follow-up and for the total study period.

The change of transition probabilities in time was evaluated, e.g. the probability of continuing ICS use of starters in the first two years (51%) of follow-up increased to more than 70% in the following years. The probabilities of different patterns of medication use were assessed: continuous use (7.7%), cumulative medication gaps (1–8 years 69.1%) and discontinuing (23.2%) during ten-year follow-up for new users. New users had lower probability of continuous use (7.7%) and more variability in ICS refill patterns than previous users (56%).

**Conclusion:**

In addition to well-established methods in epidemiology to ascertain compliance and persistence, a Markov model could be useful to further specify the variety of possible patterns of medication use within the continuum of adherence. This Markov model describes variation in behaviour and patterns of ICS use and could also be useful to investigate continuous use of other drugs applied in chronic diseases.

## Background

Chronic diseases have become more prevalent in the last decades and will be in the decades to come [1,2]. Patients suffering from these conditions need to adapt their behaviour, including taking medication, for a longer period of time, in order to decrease the risk of morbidity and mortality in the long-term. The effect of treatment on disease outcome depends for the greater part on whether sustained behaviour change has been achieved.

Medication use in chronic conditions may not always be characterized by one or several prolonged periods of drug taking in a steady dose. In some conditions like asthma, self-management allows patients, depending on their severity of disease, to adjust the timing and dosing of inhaled corticosteroids (ICS) [3-5]. Whether appropriate or not, it is very likely that the use of medication in other chronic diseases might vary similarly. Adherence rates are generally higher among patients with acute conditions compared to those suffering chronic disease, whether not symptomatic or with recurrent episodes of more frequent and more severe symptoms [6].

Adherence to pulmonary medication has shown great variability [6-9]. Although data on adherence are often reported as dichotomous variables (adherence vs. non-adherence), adherence can vary along a continuum [9-11] and only a fraction of studies have characterised medication use as such [12,13]. Discontinuation of medication has been shown to increase morbidity and mortality in asthma [14-16] and has been associated with several other diseases such as cardiovascular disorders [17,18].

Survival probabilities of continuous use are usually assessed, assuming that at the end of observation, patients were either continuous users of medication during the preceding period of follow-up or not [19]. This is inherent to single event analysis; once a patient does not fill a prescription during a certain interval, the patient is excluded from further analysis.

As the measures developed for estimating compliance from pharmacy data fail to capture the timeliness and consistency of refilling, measures to estimate persistence with medication refilling have been described. Measurement of medication persistency attempts to capture the amount of time that an individual remains on chronic drug therapy. Persistent individuals refill their medications frequently and regularly. In contrast, non-persistent individuals have a range of refilling practices or have discontinued refilling their medications completely. Instead of discontinuing treatment, which implies cessation of drug therapy with no future resumption of treatment, a patient may only have a temporary gap in treatment. Due to the relatively short duration of follow-up in most studies such temporary gaps in treatment will be considered as medication discontinuation [13]. In order to distinguish between these drug-taking behaviours with certainty, a long time horizon is necessary.

In asthma medication use had only been investigated during a maximum of two years of follow-up. Adherence (30% – 60%) and persistence rates with ICS are generally low [6,9]. The variety in results of studies on continuation of ICS use is probably due to differences in design, methods and population. Different methods for assessing continuation, comprising self-report, canister weight, physicians' estimates and pharmacy records, can explain the different results [6,9].

ICS are a type of drug that poses some methodological difficulties when analysing (non)adherence and establishing the appropriateness of medication use. Guidelines recommend to adjust the daily dose of ICS according to the severity of symptoms e.g. because of seasonal influences [3]. Classic survival analysis with discontinuation as a dichotomous measure has some limitations when aiming to describe the patterns of drug utilisation over a prolonged period of time.

We therefore aimed at characterising the different patterns of use that emerge from medication-taking behaviour in chronic disease, with a Markov chain model. With this model the probability of various patterns can be assessed assuming patient's prescription (re) filling during a fixed period. The model is explained by applying it to the (re) filling of ICS prescriptions during a maximum duration of ten years in a cohort that filled at least one ICS prescription in 1993 stratified according to new use.

## Methods

### Setting

We used data from the ongoing PHARMO record-linkage system (PHARMO RLS), which contains drug-dispensing pharmacy records from community pharmacies of 6 medium-sized cities in the Netherlands [20], covering 2% of the total Dutch population and more than 95% of all prescriptions dispensed to a particular patient [21]. Since the majority of all patients in the Netherlands are registered only with one community pharmacy, independently of prescriber, pharmacy records are virtually complete with regard to prescription drugs. Drugs are coded according to the Anatomical Therapeutic Chemical (ATC) classification. As PHARMO RLS collects data anonymously, according to current Dutch law no ethical approval for this study is required. Pharmacists that do provide dispensing data to PHARMO RLS do inform patients in general about he fact that anonymous data from their pharmacy can be used for research purposes.

### Study sample

We selected all patients that filled at least one prescription for an ICS in 1993. Of these patients the complete history of filled prescriptions from 1-1-1991 until 31-12-2002 or the end of patient observation was available. The end of follow-up is determined by PHARMO RLS either as the last date any prescription was filled by a patient at one of the pharmacies or a record of death within one of the hospitals in the linkage system.

### Statistical analysis

The probability of continuous use and the probability of cumulative gaps during follow-up were assessed by use of a Markov model (see additional file 1) for the total population adjusting for censoring. The two-sided confidence intervals for probabilities were computed applying the bootstrap method (see additional file 1) [22].

The statistical package SPLUS (version 6.2) was used for statistical analysis.

### Medication use

#### Markov model

In theory, after the filling of each prescription a patient has to decide whether or not to continue his drug therapy. This means that before filling the next prescription there is a certain probability that a patient will continue drug therapy or discontinue. In time this can result in one or several changes in refill behaviour. The patients' actions, refilling a prescription or not in several fixed periods throughout the study period, can be analysed by a Markov chain model. With the Markov model we describe it is possible to assess the probability of various patterns, instead of just continuous use and/or discontinuation. The patients' refill behaviour is characterized by the filling of at least one ICS prescription within fixed periods (calendar years) during follow-up.

In this particular Markov chain model the states are defined as the years of follow-up. A patient is considered to be in a particular state if he/she has filled at least one ICS prescription in the associated year. If a patient first filled a prescription in a particular year_*i*_, and subsequently in another year_*j*_, and not in the years between year_*i *_and year_*j*_, the patient made a transition from state " year_*i*_" to state " year_*j*_". The state "> 2002" was defined and patients with transitions into it are patients that are not censored before the end of the study (31-12-2002) and of which no assessment of medication use can be performed due to the end of (study) follow-up. The transitions after the end of follow-up are unknown. Transitions from each state are the filling of a prescription in the next calendar year, in one of the other following years or in none of the years while under observation. For each state several transitions from and into another state are possible. As the filling of at least one prescription for an ICS is an inclusion criterion for the cohort all patients are present in the state year_93_. The first state, 1993, has no transition into it. The number of possible transitions for each state depends on the number of remaining years of follow-up after year_*i*_. The later the year under consideration, the less the number of possible transitions becomes. For example, the first year of follow-up, 1993, has the most number of achievable transitions to another state; 10 and the last year of follow-up, 2002, has the least; 1. Clearly the state ">2002" has no transitions to other states.

As stated before transition probabilities can be assessed for the transition from state_*i *_into state_*j*_, to be more precise year_*i *_into year_*j*_. To do so, a period of at least two consecutive years is necessary. From the state "2002" patients can only have transitions into ">2002" due to the end of follow-up. A first-order Markov chain model like the one we describe (see appendix) implies that the presence in a particular state only depends on the presence in the directly preceding state but is independent of all former states. This means that the probability of having filled at least one ICS prescription in 1996 and subsequently filling at least one prescription for an ICS in one of the following years, for instance 1997, are conditional to having filled at least one prescription in a preceding year. To have a transition from 1996 to another state, e.g. to refill a prescription in one of the years after 1996, a patient should have filled at least one prescription in one of the years before 1996. The possible transitions into the state, 1996, are 1) year_93_→year_96 _(the previous prescription(s) filled in 1993), 2) year_94_→year_96 _(the previous prescription(s) filled in 1994), 3) year_95_→year_96_, (the previous prescription(s) filled in 1995). E.g. in possibility 2) filling at least one prescription in 1997 given 1996; 1994; 1993, the transition from year_96_→year_97 _is conditional only to the transition from year_94_→year_96 _regardless of the transition into year_94_.

The transition matrix gives an overview of the probabilities for all possible transitions throughout the study period. The sum of all possible transition probabilities from a certain state is 1. As the states in this model are defined as subsequent time periods, the transition matrix is a visual description of possible behaviour and change in time. It is therefore possible to detect a change in time of a certain transition probability, e.g. for the filling of the next prescription in the immediately following year.

The occurrence of continuing ICS use can be affected by several factors, possibly leading to confounding. In order to adjust for potential confounding, a stratified analysis according to these factors can be performed. An important confounder is the history of patients at the start of the study. Previous users are "survivors" of the early period of pharmacotherapy and are likely to have a higher probability of continuous use [23]. Therefore new and previous users were defined based on the use of ICS in 1991 and/or 1992.

#### Continuous use

**Figure 1:**
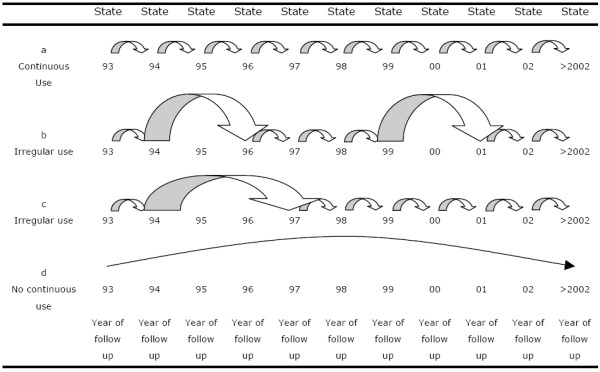
Examples of patterns of medication use during follow-up. In these examples patients are observed during maximum follow-up. 1a A patient with continuous use throughout the study period. 1b A patient with periods of ICS prescription refill in subsequent years of follow-up and two separate years without a refill prescription; cumulative medication gap of two years. 1c A patient with periods of ICS prescription refill in subsequent years of follow-up and two consecutive years without a refill prescription; cumulative medication gap of two years. 1d A patient, that did not refill an ICS prescription during follow-up while still under observation.

The transition probabilities for the filling of at least one ICS prescription for all different years of follow-up, can be combined (see appendix) in order to assess the probability of a particular "route" throughout the total period of follow-up, such as continuous use of ICS (Figure 1). In the content of this study continuous use was defined as the filling of at least one prescription for an ICS in each year of follow-up (calendar year) (Figure 1a). A sensitivity analysis was performed with more strict definitions of at least two and at least three prescriptions filled per year.

#### Medication gaps

Some of the conceivable patterns of patients' variable prescription refill behaviour are shown in Figure 1. The distribution of patterns with different numbers of cumulative gaps for the follow-up period can be obtained by use of the transition matrix.

Medication gaps were defined as "calendar years without any prescription for an ICS". The occurrence of a gap of one year in the period [1993; 1995] would result in the filling of the next prescription for an ICS in 1995 after 1993, which corresponds to the transition probability *P*_93→95 _(see appendix). The probabilities of cumulative gaps were assessed during the total duration of follow-up (see appendix), for example the probability of a gap of one year ***T***^9 ^*[1993, >2002] *- ***T***^8 ^*[1993, >2002]*, cumulative gap of five years, and so on. Gaps could be in consecutive years or spread over several years. A patient with a cumulative gap duration of two years could have filled prescriptions for ICS in 1993 and for instance subsequently in each year during follow-up, except for 1995 and 2000 (Figure 1b). Another patient could have the same cumulative gap duration with a prescription in each year during follow-up, except for 1995 and 1996 (Figure 1c).

## Results

In 1993, a total of 9,234 patients filled at least one prescription for an ICS. Of all patients receiving ICS in 1993, 5481 (59.4%) were characterised as previous users of ICS as they filled at least one prescription in 1991 or 1992. Based on this classification, 3367 (36.5%) patients were characterised as new users of ICS. The history preceding 1993 was not available for 386 patients (4.1%), as these were not observed during the two previous years. From this population, 57.0% was followed for ten years, the maximum duration of follow-up. The mean follow-up was 7.7 years (Table 1).

**Table 1:** Baseline characteristics of the study population in intitial year of study (Undetermined previous use due to insufficient medication history prior to 1993 for 386 (4.2%) patients)

**At least one ICS prescription filled in 1993**	New users	Previous users (≥ 1 prescription in 1991 or 1992)
	(% or range)	(% or range)

All patients	3,367 (36.5)	5,481 (59.4)
Male (%)	1,629 (48.4)	2,813 (51,3)
Mean age (yrs)	43.9 (0–94)	50.7 (2–99)
Mean follow-up (yrs)	7.7 (0.04–10.3)	7.7 (0.02–10.3)
Mean number of ICS prescriptions filled in 1993	2.2 (1–28)	3.6 (1–25)

### Continuous use

**Figure 2:**
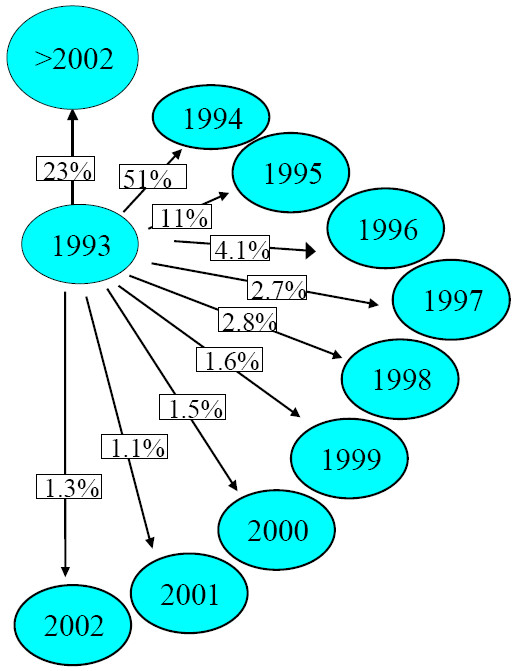
Transition probabilities from one particular state, 1993, to all other possible states for new users are shown. The transition probability of filling at least one ICS prescription in 1994, given filling at least one prescription in 1993, P_93→94_, is 51%. One of the possible transitions is *"1993" → "after end of follow-up"*.

**Figure 3:**
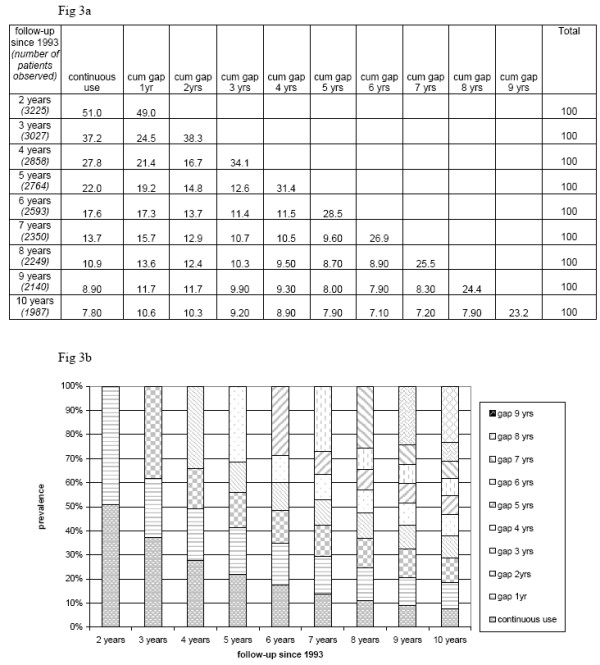
3a For new users, the probabilities of continuous use, gaps and discontinuation in the period that has elapsed until a particular year of follow-up. 3b For new users, for each year of follow-up the proportion of patients with irregular ICS use (medication free periods) and continued ICS use in the period of follow-up that has elapsed until then are shown.

**Figure 4:**
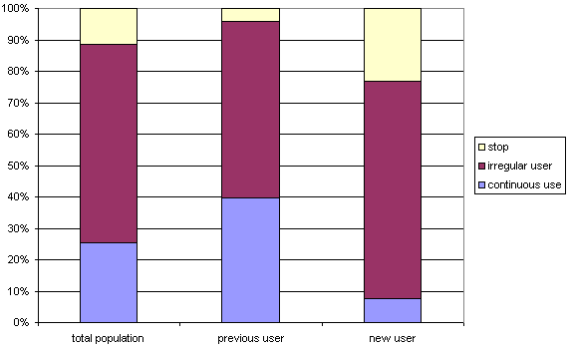
The probability of gaps, medication free periods of several lengths, in the total population and stratified for new and previous use.

Probabilities of continuous use and medication free periods were assessed for the total population and stratified according to new use. Previous users had a higher probability of continuous use, 39.8% (95% C.I. 22.1–26.0%) than new users, 7.8% (95% C.I. 6.8–8.7%). The probabilities obtained for new users are presented in table 3 and Figures 2, 3, 4.

The probabilities of continuous use hardly varied with more strict definitions of continuous use. The probabilities of continuous use for new users with a definition of at least two or at least three prescriptions per year were respectively 10.2% (95% C.I. 8.6–11.8) and 10.2% (95% C.I. 8.6–11.8) (data not shown).

For each year the transition probabilities, assessed by Markov analysis, are presented in Table 1. The transition probabilities for any of the years of follow-up, are printed in the rows. To clarify the outcome, transition probabilities for only 1993 are shown in Figure 2. The transition probabilities for the refilling of a prescription in any of the subsequent years of follow-up for every state can be written out in the same way. As stated earlier the probabilities of transitions for a particular event from different states can be combined (see appendix) in order to assess the probability of a particular "route" during follow-up.

In 1994, year 1 and 2 of follow-up have elapsed and the maximum duration of use that can be obtained is two years, 37.2% of the patients filled a prescription in both, 1993 and 1994. By combining (see appendix) the transition probability of the filling of a prescription in 1994 (*p*_93→94_) with the probability of filling the next prescription in 1995 (*p*_94→95_) the prevalence of patients that are continuous users in 1994 (0.51*0.73*100% = 37.2%) is obtained. Based on the first order Markov model, which assesses conditional transition probabilities, a patient that was a continuous user in 1996, has been a continuous user in every previous year (*p*_93→94 _* *p*_94→95 _*p_95→96_). In 1996 (0.51*0.73*0.75*100% =) 27.9% filled a prescription in every preceding year.

Figure 3 illustrates that patients do not refill a prescription for an ICS in each year of follow-up and in time this results in variable patterns.

As explained earlier, the population was stratified according to previous and new use of ICS. The probability after ten years of follow-up was 39.7% (95% C.I. 38.0–41.3) for previous users and was substantially lower for new users, 7.8% (95% C.I. 6.8–8.7). It is possible to investigate whether this lower probability of continuous use in 1993 is constant and sustained for each state throughout the study. In the first year of follow-up 85.1% (95% C.I. 84.1–86.0) of the previous users continued ICS use as opposed to 51.0% (95% C.I. 49.4–52.9) of the new users. After the first three years the probability increased to 79% and stabilises (Table 2, printed in bold).

**Table 2:** Matrix of transition probabilities. The states in this model are defined as the years of follow-up

***T *=**		1993	1994	1995	1996	1997	1998	1999	2000	2001	2002	>2002	Total
	1993	0	**0.51**	0.11	0.04	0.03	0.03	0.02	0.01	0.01	0.01	0.23	1
	1994	0	0	**0.73**	0.08	0.05	0.02	0.02	0.01	0.004	0.01	0.09	1
	1995	0	0	0	**0.75**	0.10	0.03	0.01	0.01	0.01	0.01	0.08	1
	1996	0	0	0	0	**0.79**	0.08	0.02	0.02	0.01	0.01	0.07	1
	1997	0	0	0	0	0	**0.79**	0.08	0.03	0.01	0.01	0.07	1
	1998	0	0	0	0	0	0	**0.78**	0.09	0.03	0.02	0.09	1
	1999	0	0	0	0	0	0	0	**0.80**	0.07	0.03	0.10	1
	2000	0	0	0	0	0	0	0	0	**0.81**	0.08	0.10	1
	2001	0	0	0	0	0	0	0	0	0	**0.87**	0.13	1
	2002	0	0	0	0	0	0	0	0	0	0	1	1
	>2002	0	0	0	0	0	0	0	0	0	0	1	1

### Medication gaps

Markov analysis was used to ascertain the probability of medication free periods of several lengths. Of the patients observed in 1993, the year of start for new users, 51.0% (95% C.I. 49.4–52.9) filled at least one prescription in the following year (Table 3 top row, Figure 2). In this first year, 1993, new users had a probability of 11% (95% C.I. 9.7–11.8) for a gap of exactly one year, the filling of the next prescription for an ICS in 1995 (Table 3 top row, Figure 2).

In addition to a lower probability of continuous use, new users tend to have medication free periods of three years or longer (69.1%), more frequently than previous users (56%) (Figure 4). Besides the filling of a prescription in each year (continuous use), not in all years of follow-up (gaps), not refilling a prescription (discontinuation) was also considered one of the possible patterns of medication use. The probability of discontinuation of ICS refilling after 1993 throughout the study is higher in new users (23.2%) than in previous users (4.3%). These findings illustrate that new users have more variability in patterns of ICS use during a period of ten years.

## Discussion

The Markov chain model that was designed enables to describe the variable patterns that emerge from refilling ICS prescriptions during a period of maximum possible follow-up of ten years among patients that filled at least one ICS prescription in 1993 in the PHARMO RLS. New users of ICS in 1993 have a lower probability of continuous use and more variability in patterns of ICS use than previous users. This can be completely attributed to the finding that the probability of continuing treatment into the next year increased for new users from 51% in the first two years to more than 70% in the following years. New use and long-term persistence have, to our knowledge, not been well investigated among ICS users, but similar differences between new and previous users have also been shown in other drug treatment regimens [23].

Chronic diseases require long-term adjustments in patients' behaviour, such as medication taking behaviour. Potential consequences of medication non-adherence include disease progression in chronic illness, and the subsequent need for more aggressive treatments, which further increases the risk of (drug-induced) illness [14-16,18]. In chronic conditions, especially when not symptomatic, motivation might change over time adding substantially to the variability in adherence to preventer medication, leading to worsening of the condition. In our study the probability of continuing ICS use of starters in the first two years was lowest and increased to approximately the same probability as for previous users in the following years. Although "survival" of a group of patients with higher persistence was expected [23,24] this is not observed in all studies on persistence in new users [25].

### Clinical implications

It is important to realise that patients might discontinue for several reasons. Patients using ICS, however most frequently do not discontinue entirely but use their medication intermittently or during annual periods often based on seasonal variety of symptoms. When initiating therapy, patient and physician should therefore agree on the intended duration of use and subsequently evaluate the experience of symptoms and the use of ICS. By reviewing dispensing data pharmacists could assist physicians in monitoring adherence. Patients' previous experiences with a specific drug treatment should be discussed as these experiences might influence future behaviour.

### Research implications

Often medication use, not only in asthma, is studied only during a short period of time as a dichotomous outcome [7-9,11-13,24,25] and not often in relation to clinical outcome [26,27]. The necessary level of adherence for treatment effectiveness depends on the drug and the disease, rendering meaningless any arbitrary distinction between "adherent" and "non-adherent". Therefore, there is a need for describing medication use as a continuum and the varying patterns in time. The analysis of drug dispensing records often results in aggregated population characteristics, which do not clarify individual changes in medication. Consequently deviant behaviour is not easily detected. As patient profiles and drug-use patterns over time are important determinants of treatment outcomes, a method that characterizes individual behaviour over a longer period of time is needed.

In the frequently applied two state model a patient "disappears" from the analysis after the (first) event. In the Markov model described a patient stays in the analysis after the first event, which gives more insight into different behaviour patterns of long-term medication use. Moreover, a Markov model can be intuitively graphically understood which is a clear advantage for interpretational purposes.

Detailed information on dosing schedules and differences in type and number of prescription drugs used can further characterise drug-use patterns. This detailed information can also help to understand patient behaviour. Furthermore, drug related characteristics such as initiation, refilling, switching and discontinuation could be analysed [28]. In this Markov model the transition states were defined as a certain number of prescriptions filled per year to describe refill behaviour in patients using asthma preventer therapy. Additionally, states can be defined by applying previously described methods for assessing drug exposure, such as single or multiple-interval measures of medication gaps [12,13]. Thus combining accepted methods in epidemiology and a relatively new type of modelling, which previously has been applied to great extent in economics and has not been used often in clinical epidemiology.

## Conclusion

In addition to well-established methods in epidemiology to ascertain compliance and persistence, a Markov model could be useful to further specify the variety of possible patterns of medication use within the continuum of adherence. This Markov model describes variation in behaviour and patterns of ICS use and could also be also be used to investigate patterns of use of other drugs applied in chronic diseases.

## Competing interests

JR is part time professor at the University of Utrecht and member of the board of GlaxoSmithKline in the Netherlands. All other authors declare that they have no competing interests.

## Authors' contributions

All authors formulated the research question and design of the study. TM carried out the study. SB programmed the Markov model, which was designed specifically for this study. SB, Mbo, MBr, J-WL, JR and HL advised on the analyses. TM drafted and revised the manuscript. All authors critically reviewed draft versions of the manuscript. All authors read and approved the final manuscript.

## Pre-publication history

The pre-publication history for this paper can be accessed here:



## Supplementary Material

Additional File 1 Appendix Statistical description of the Markov model designed for the analysis of ICS use in this study. Click here for file

## References

[B1] (2003). Diet, nutrition and the prevention of chronic diseases.

[B2] (2002). WHO strategy for prevention and control of chronic respiratory diseases.

[B3] GINA (2005). Global Initiative for Asthma: Global strategy for asthma management and prevention.

[B4] Powell H, Gibson PG (2004). Initial starting dose of inhaled corticosteroids in adults with asthma: a systematic review. Thorax.

[B5] Boulet LP (2004). Once-daily inhaled corticosteroids for the treatment of asthma. Curr Opin Pulm Med.

[B6] Osterberg L, Blaschke T (2005). Adherence to medication. N Engl J Med.

[B7] Cochrane GM, Horne R, Chanez P (1999). Compliance in asthma. Respir Med.

[B8] Cochrane MG, Bala MV, Downs KE, Mauskopf J, Ben-Joseph RH (2000). Inhaled corticosteroids for asthma therapy: patient compliance, devices, and inhalation technique. Chest.

[B9] Breekveldt-Postma NS, Gerrits CM, Lammers JW, Raaijmakers JA, Herings RM (2004). Persistence with inhaled corticosteroid therapy in daily practice. Respir Med.

[B10] Rudd P, Ahmed S, Zachary V, Barton C, Bonduelle D (1992). Issues in patient compliance: the search for therapeutic sufficiency. Cardiology.

[B11] Rudd P, Byyny RL, Zachary V, LoVerde ME, Mitchell WD, Titus C, Marshall G (1988). Pill count measures of compliance in a drug trial: variability and suitability. Am J Hypertens.

[B12] Steiner JF, Prochazka AV (1997). The assessment of refill compliance using pharmacy records: methods, validity, and applications. J Clin Epidemiol.

[B13] Sikka R, Xia F, Aubert RE (2005). Estimating medication persistency using administrative claims data. Am J Manag Care.

[B14] Donahue JG, Weiss ST, Livingston JM, Goetsch MA, Greineder DK, Platt R (1997). Inhaled steroids and the risk of hospitalization for asthma. Jama.

[B15] Suissa S, Ernst P, Kezouh A (2002). Regular use of inhaled corticosteroids and the long term prevention of hospitalisation for asthma. Thorax.

[B16] Lanes SF, Garcia Rodriguez LA, Huerta C (2002). Respiratory medications and risk of asthma death. Thorax.

[B17] Degli Esposti E, Sturani A, Degli Esposti L, Macini PL, Falasca P, Valpiani G, Buda S (2001). Pharmacoutilization of antihypertensive drugs: a model of analysis. Int J Clin Pharmacol Ther.

[B18] Psaty BM, Koepsell TD, Wagner EH, LoGerfo JP, Inui TS (1990). The relative risk of incident coronary heart disease associated with recently stopping the use of beta-blockers. Jama.

[B19] Rothman KJ (2002). Measuring disease occurrence and causal effects. Epidemiology An introduction.

[B20] Herings RM, Bakker A, Stricker BH, Nap G (1992). Pharmaco-morbidity linkage: a feasibility study comparing morbidity in two pharmacy based exposure cohorts. J Epidemiol Community Health.

[B21] Herings RM (1993). PHARMO: A record linkage system for postmarketing surveillance of prescription drugs in the Netherlands.. Utrecht Institute for Pharmaceutical Sciences.

[B22] Efron B, Tibshirani RJ (1993). An introduction to the bootstrap.

[B23] Ray WA (2003). Evaluating medication effects outside of clinical trials: new-user designs. Am J Epidemiol.

[B24] Van Wijk BL, Klungel OH, Heerdink ER, de Boer A (2005). Rate and determinants of 10-year persistence with antihypertensive drugs. J Hypertens.

[B25] Mantel-Teeuwisse AK, Goettsch WG, Klungel OH, de Boer A, Herings RM (2004). Long term persistence with statin treatment in daily medical practice. Heart.

[B26] van der Elst ME, Cisneros-Gonzalez N, de Blaey CJ, Buurma H, de Boer A (2003). Oral antithrombotic use among myocardial infarction patients. Ann Pharmacother.

[B27] Bouvy ML, Heerdink ER, Leufkens HG, Hoes AW (2003). Patterns of pharmacotherapy in patients hospitalised for congestive heart failure. Eur J Heart Fail.

[B28] Leufkens HG (2002). Pharmacoepidemiological modelling: Markov models of antibiotic use in patients with diabetes. Neth J Med.

