# Supplementary material for: Distinguishing patterns in the dynamics of long-term medication use by Markov analysis: beyond persistence
Source: BMC Health Serv Res. 2007 Jul 10;7:106. doi: 10.1186/1472-6963-7-106 (PMC1959200; doi:10.1186/1472-6963-7-106)
Supplement: Additional File 1 — Appendix Statistical description of the Markov model designed for the analysis of ICS use in this study. [file 1472-6963-7-106-S1.doc]

## Appendix

A general homogeneous Markov chain model for epidemiological longitudinal studies of appearance of certain event was designed to analyse patterns of drug use. The states of the Markov chain represent the years when the prescription(s) can be filled. The transition matrix of the process consists of transition probabilities and is as follows:

|  |  | 1993 | 1994 | 1995 | ... | 2002 | >2002 | Total |
| --- | --- | --- | --- | --- | --- | --- | --- | --- |
|  | 1993 | 0 | *p_93→94_* | *p_93→95_* | ... | *p_93→02_* | *p_93→>02_* | 1 |
|  | 1994 | 0 | 0 | *p_94→95_* | ... | *p_94→02_* | *p_94→>02_* | 1 |
| ***T* =** | 1995 | 0 | 0 | 0 | ... | *p_95→02_* | *p_95→>02_* | 1 |
|  | ... | ... | ... | ... | ... | ... | ... | ... |
|  | 2001 | 0 | 0 | 0 | ... | *p_01→02_* | *p_01→>02_* | 1 |
|  | 2002 | 0 | 0 | 0 | ... | 0 | 1 | 1 |
|  | >2002 | 0 | 0 | 0 | ... | 0 | 1 | 1 |

A transition probability from state *i* to state *j* ( notation: *p_i j_* ) is a conditional probability that the prescription in year *j* is filled given the previous time of the filling of a prescription was year *i*. The estimates of the transition probabilities based on censored observations are

*p_i i+1_*  = *x_i i+1_*  / *n_i i+1_*

*p_i j_*  = ( *x_i j_*  / *n_i j_*  ) (1− *p_i i+1_*  – ... − *p_i j−1_* ) ,

for *i, j* ∈ { 1993, 1994, ..., 2002, >2002}, *j*>*i*,

where *x_i j_*  is the number of patients who used the medication in years *i* and *j* and not in the period between these years; *n_i j_*  is the number of patients observed in year *j* that used the drug in year *i* and did not take the medication until year *j*.

To describe the patterns of drug use, we apply Chapman-Kolmogorov equation to derive the following estimates of the probabilities:

| Estimates of the probabilities that a patient observed in period from 1993 until 2003 did not use the medication during | 0 years | is | ***T ^10^*** *[1993, >2002] -* ***T ^9^*** *[1993, >2002]* |
| --- | --- | --- | --- |
|  | 1 years |  | ***T ^9^*** *[1993, >2002] -* ***T ^8^*** *[1993, >2002]* |
|  | 2 years |  | ***T ^8^*** *[1993, >2002] -* ***T ^7^*** *[1993, >2002]* |
|  | ... |  | ... |
|  | 7 years |  | ***T ^3^*** *[1993, >2002] -* ***T ^2^*** *[1993, >2002]* |
|  | 8 year |  | *(****T*** × ***T****)[1993, >2002] -* ***T****[1993, >2002]* |
|  | >= 9 years |  | ***T****[1993, >2002]* |

| Estimates of the probabilities that a patient observed in period from 1993 until 1993 + j  did not use the medication during | 0 years | is | ***_N N_***  ***Σ T ^j^[1,i] - Σ T ^j-1^[1,i]***  ***^i=j+1 i=j+1^*** |
| --- | --- | --- | --- |
|  | 1 years |  | ***_N N_***  ***Σ T ^j-1^[1,i] - Σ T ^j-2^[1,i]***  ***^i=j+1 i=j+1^*** |
|  | 2 years |  | ***_N N_***  ***Σ T ^j-2^[1,i] - Σ T ^j-3^[1,i]***  ***^i=j+1 i=j+1^*** |
|  | ... |  | ... |
|  | ( j -3 ) years |  | ***_N N_***  ***Σ T ^3^[1,i] - Σ T ^2^[1,i]***  ***^i=j+1 i=j+1^*** |
|  | ( j -2) years |  | ***_N N_***  ***Σ (T × T)[1,i] - Σ T[1,i]***  ***^i=j+1 i=j+1^*** |
|  | >= ( j -1) years |  | ***_N_***  ***Σ T[1,i]***  ***^i=j+1^*** |

The two-sided 95% bootstrap percentile confidence intervals for transition probabilities and probabilities of gaps were computed using 200 replications. Bootstrap samples were obtained by random sampling patients (with replacement) from the population under consideration, i.e. the entire Markov chains associated with the corresponding patients.
